# Employment conditions as barriers to the adoption of COVID-19 mitigation measures: how the COVID-19 pandemic may be deepening health disparities among low-income earners and essential workers in the United States

**DOI:** 10.1186/s12889-022-13259-w

**Published:** 2022-05-02

**Authors:** Ariadna Capasso, Sooyoung Kim, Shahmir H. Ali, Abbey M. Jones, Ralph J. DiClemente, Yesim Tozan

**Affiliations:** 1 Department of Social and Behavioral Sciences, School of Global Public Health, New York University, New York, USA; 2 Department of Health Policy and Management, School of Global Public Health, New York University, New York, USA; 3 Department of Epidemiology, School of Global Public Health, New York University, New York, USA; 4 Global and Environmental Health Program, School of Global Public Health, New York University, 708 Broadway, New York, 10003 USA

**Keywords:** COVID-19, Essential workers, Risk of infection, Health Belief Model, Employment conditions, Economic precarity, Precarious employment, Health disparities, Social determinants of health

## Abstract

**Background:**

The COVID-19 pandemic has disproportionately impacted economically-disadvantaged populations in the United States (US). Precarious employment conditions may contribute to these disparities by impeding workers in such conditions from adopting COVID-19 mitigation measures to reduce infection risk. This study investigated the relationship between employment and economic conditions and the adoption of COVID-19 protective behaviors among US workers during the initial phase of the COVID-19 pandemic.

**Methods:**

Employing a social media advertisement campaign, an online, self-administered survey was used to collect data from 2,845 working adults in April 2020. Hierarchical generalized linear models were performed to assess the differences in engagement with recommended protective behaviors based on employment and economic conditions, while controlling for knowledge and perceived threat of COVID-19, as would be predicted by the Health Belief Model (HBM).

**Results:**

Essential workers had more precarious employment and economic conditions than non-essential workers: 67% had variable income; 30% did not have paid sick leave; 42% had lost income due to COVID-19, and 15% were food insecure. The adoption of protective behaviors was high in the sample: 77% of participants avoided leaving home, and 93% increased hand hygiene. Consistent with the HBM, COVID-19 knowledge scores and perceived threat were positively associated with engaging in all protective behaviors. However, after controlling for these, essential workers were 60% and 70% less likely than non-essential workers, who by the nature of their jobs cannot stay at home, to stay at home and increase hand hygiene, respectively. Similarly, participants who could not afford to quarantine were 50% less likely to avoid leaving home (AOR: 0.5; 95% CI: 0.4, 0.6) than those who could, whereas there were no significant differences concerning hand hygiene.

**Conclusions:**

Our findings are consistent with the accumulating evidence that the employment conditions of essential workers and other low-income earners are precarious, that they have experienced disproportionately higher rates of income loss during the initial phase of the COVID-19 pandemic and face significant barriers to adopting protective measures. Our findings underscore the importance and need of policy responses focusing on expanding social protection and benefits to prevent the further deepening of existing health disparities in the US.

**Supplementary Information:**

The online version contains supplementary material available at 10.1186/s12889-022-13259-w.

## Introduction

Socio-economic status (SES), defined by constructs such as education, occupation, and income [1], has long been established as a fundamental cause of disease [2]. Mounting evidence suggests that the ongoing COVID-19 pandemic is widening the existing socio-economic disparities in the United States (US) and elsewhere by taking a disproportionate toll on the health and wellbeing of people with lower SES [3–9].

Some sectors of the population bore the double brunt of economic strain and COVID-19 risk. During the early months of the COVID-19 pandemic, low-income workers disproportionately experienced job losses [6]. Concurrently, job loss and economic insecurity were associated with higher levels of stress, anxiety, and depressive symptoms [8]. Due to their employment, essential low-wage workers, such as grocery store workers and nurse health aides, are at high risk for COVID-19 exposure [6]. Indeed, studies have documented excess COVID-19 mortality among workers in such occupational sectors, such as agriculture, manufacturing, and healthcare support [10, 11]. The risk is compounded by poverty, which may increase risk of infection due to unavoidable physical crowding and poor access to sanitation [9].

US studies have documented growing SES and health disparities during the COVID-19 pandemic [3–9]. These disparities may be partially attributable to the absence of universal health coverage and sick leave policies, coupled with the impact of employment conditions on COVID-19-related health outcomes [6, 8, 12, 13]. In particular, precarious job conditions, characterized by lack of fixed income and health and social benefits, as well as financial insecurity [14, 15], are likely to be critical, yet understudied, factors explaining the disproportionate burden of COVID-19 on the working poor. Further, the current evidence on the role of employment conditions as barriers or facilitators to adopting recommended COVID-19 protective behaviors is not robust.

**Fig. 1:**
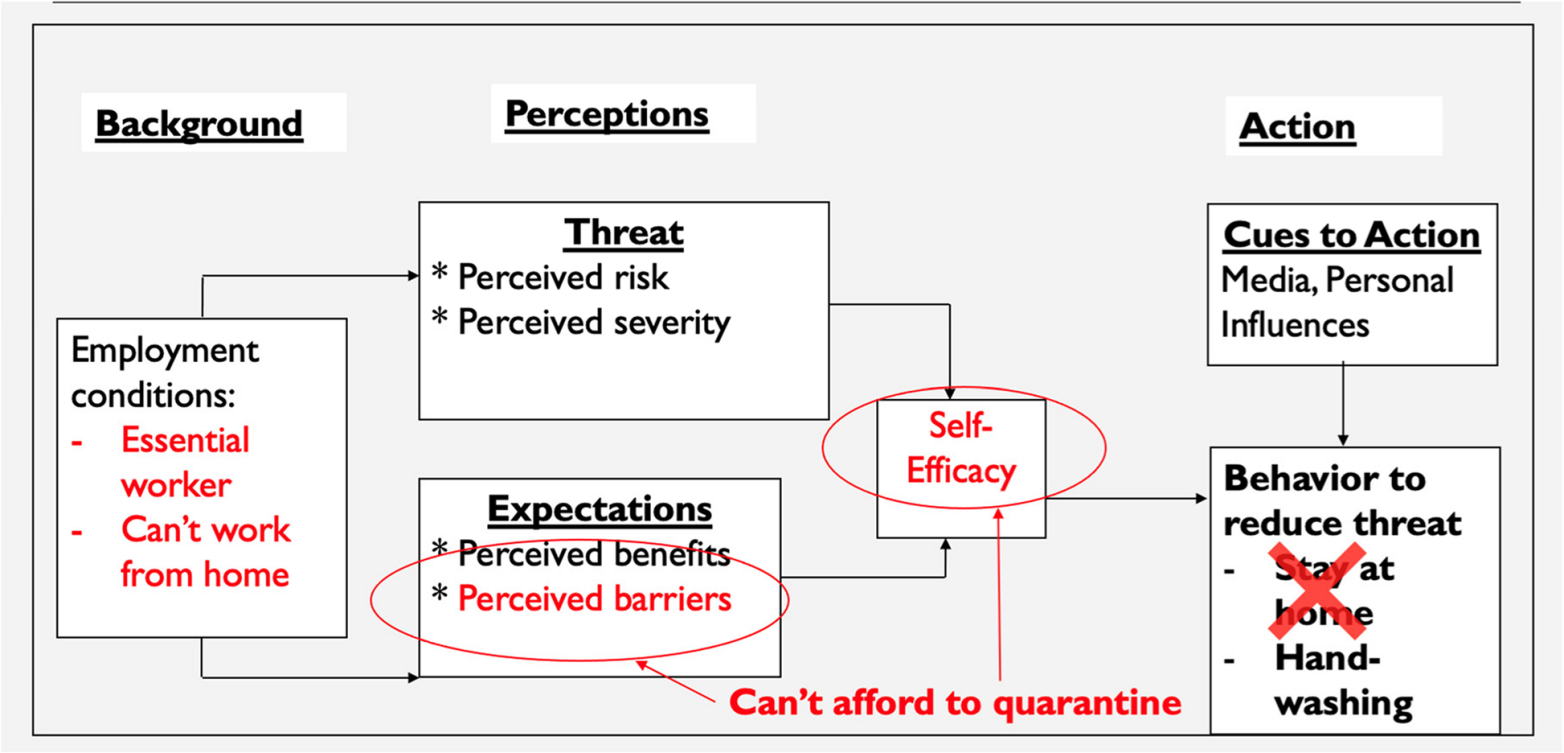
Conceptual model depicting how certain employment conditions may be barriers to COVID-19 preventive behaviors

This study investigated the potential mechanisms through which disparities in SES, with a specific focus on employment conditions and income, influence people’s ability to engage in COVID-19 protective behaviors. To do so, we analyzed data collected from a sample of currently working adults in the US and used the Health Belief Model (HBM), a widely used model informing behavior change interventions, as a framework to guide our analytic model [16]. The HBM posits that health-protective behaviors are influenced by perceived disease threat—which is composed of perceived susceptibility and severity—, self-efficacy to adopt protective behaviors, and the perceived benefits and barriers to engaging in these behaviors (See Fig. 1) [17]. While the predictive power of the HBM and its constructs are still under discussion [14], the HBM provides a useful theoretical framework to explain the pathways from knowledge, perceptions, and beliefs to health-related actions.

Our study investigates the following hypotheses:

- *Hypothesis 1: Knowledge of recommended COVID-19 protective behaviors and COVID-19 threat perception are positively associated with engagement in such protective behaviors, such as frequent handwashing.*
- *Hypothesis 2: Adoption of protective behaviors related to social distancing, such as staying at home, is determined by employment conditions. Specifically, from the perspective of the HBM, employment conditions impede self-efficacy to engaging in certain COVID-19 protective behaviors but not others.*

## Methods

### Participant recruitment and survey administration

The study methodology is described in detail elsewhere [18]. In brief, an online survey on Qualtrics (Provo, UT) targeted English-speaking social media users via an advertisement campaign on Facebook and affiliated platforms. Eligibility criteria included being at least 18 years of age and a resident of the US. A list of COVID-19 resources from the US Centers for Disease Control and Prevention (CDC) and the World Health Organization (WHO) were shared with ineligible participants and respondents after survey completion. Social media platforms offer a feasible and low-cost platform for rapid participant recruitment in health and social sciences research [19, 20]. Recruitment occurred from April 16–21, 2020 (*N* = 5,062). Respondents received no incentives for participation. The current analytic sample comprised 2,845 respondents who reported being currently employed and identified themselves as working full-time (*n* = 2067), part-time (*n* = 402), self-employed (*n* = 371), or in the military (*n* = 5). The [redacted] Institutional Review Board approved the study as exempt and waived the need for explicit written or oral consent.

### Survey measures

Questionnaire development was guided by the HBM [21], a model widely used to inform behavior change interventions and applied in prior research on infectious disease epidemics [22–25], and the WHO’s COVID-19 behavioral insights survey tool and guidance document [26].

*Knowledge of COVID-19 protective behaviors* was assessed by two binary (True/False) items, asking respondents whether they could protect themselves from being infected with COVID-19 by “Washing your hands frequently with soap and water” and “Stopping going to school/work.” Whereas as the pandemic evolved, resuming day-to-day activity with a mask became the norm, this was not the case in the early stages of the pandemic when many locations instituted stay-at-home orders. Thus, these questions were intended to capture handwashing and social distancing, two of the primary COVID-19 prevention measures promoted at the time of survey implementation (April 2020).

The HBM deconstructs disease threat as perceived susceptibility or a person’s subjective perception of the risk of acquiring an illness or disease and perceived severity of the seriousness of contracting an illness or disease [21]. As recommended [21], the two constructs were measured separately, as follows: *Perceived COVID-19 risk* was assessed by the question “On a scale from 0-10, what do you think is your risk of getting infected with Coronavirus?” *Perceived COVID-19 severity* was assessed by a single item, “On a scale from 0-10, if you were infected with Coronavirus, how severe do you think it would be?” On this scale, 0 signifies very low perceived risk or severity, and 10 signifies very high perceived risk or severity.

The socio-demographic information of respondents included: sex, age (18-39 years; 40-59 years, ≥60 years), marital status (single/separated/divorced; married/partnered), race (coded as non-Hispanic white and non-white because of low numbers among minority groups), educational attainment (college degree or above; some college or below), type of residence (urban; suburban; rural), US Census region [27] (coded based on reported state of residence as Northeast, Midwest, South, and West).

An *economic precarity index* was developed based on the sum of the following binary items: 1) annual household income (≥90,000; ≥50,000-<90,000; <50,000); 2) variable income (in response to “How do you get paid?”; salaried employees were coded as fixed income and those being paid per hour, per job or based on other arrangements, as variable income); 3) no paid sick leave (coded ‘yes’ if employers did not offer paid sick leave); 4) no health insurance (coded ‘yes’ if a respondent reported not having any type of health insurance); 5) lost income due to COVID-19 (yes/no); and 6) food insecurity in the last 3 months (assessed with the six-item USDA Household Food Security Survey Module [28] adapted to gauge food insecurity since the start of the COVID-19 pandemic in the US, where summed scores under 2 were considered as food secure, and scores from 2 to 6 as food insecure). The index scores ranged from 0 to 6, with higher scores indicating higher precarity. Income was recoded as ≥50,000 = 0 and <50,000 = 1 for the index; this cutoff was selected for comparability with other national surveys [29].

To assess if economic precarity associated with engaging in certain protective behaviors but not others, an “essential worker” variable was created by coding all respondents who answered ‘Yes’ to either of two questions: 1) “Are you considered an essential worker (i.e., do you have to go into work when others in your community have been asked to stay at home)?” or 2) “If you were required to remain at home because of a quarantine or work closure, would you be able to do at least part of your job from home?” In addition, a separate binary measure was used to assess whether people thought that they could afford to quarantine if mandated to do so. Within the framework of the HBM, we used these two measures as a proxy for self-efficacy to engage in protective behaviors related to physical distancing (e.g., avoid leaving home), but not in behaviors related to hygiene (e.g., handwashing, cleaning, and disinfecting more).

The primary outcome measures were four binary (Yes/No) measures assessing whether respondents endorsed the following four COVID-19 protective behaviors in response to the question “To protect myself from getting infected with Coronavirus, I ...”: 1) “Avoided leaving home except for food or medical supplies”; 2) “Started using hand-sanitizer and/or washing my hands more often”; 3) “Started cleaning and/or disinfecting things that I might touch (e.g., doorknobs, phone)”; and 4) “Avoided seeking medical or dental care for other [non COVID-19] health concerns.” The first three behaviors were recommended by the CDC to protect against COVID-19 infection at the time of survey administration. The fourth behavior was recommended by some policymakers at the beginning of the pandemic for the double purpose of reducing the risk of COVID-19 exposure at medical centers and prioritizing strained healthcare resources for the treatment of COVID-19 cases.

### Statistical analysis

Descriptive statistics were calculated for the total sample and by essential worker status. Polychoric correlations were estimated to assess the relationships between the variables that make up the economic precarity index. Hierarchical generalized linear models (GLM) [30] with robust estimators estimated the odds ratios and 95% confidence intervals for the four COVID-19 protective behaviors. In the first step, we included all the socio-demographic variables that were identified as significant in bivariate analysis; in the second step, we included one knowledge variable at a time; in the third step, we added perceived COVID-19 risk and severity; and, in the final step, we included the economic precarity index, essential worker status, and not being able to afford to quarantine. The knowledge question on handwashing was removed from multiple regression analysis because the models did not converge due to zero cells and low numbers in some cells (almost 100% of the sample reported that handwashing was protective of COVID-19). All models accounted for clustering due to state of residency and were estimated specifying the ‘binary’ family and the ‘logit’ link. Comparison of model fit was conducted with likelihood ratio tests of nested models. Adjusted odds ratios were graphed with ‘coefplot.’ Complete case analysis was used because most variables had no missing data, and sex and educational attainment had less than 1% missing values. Paid sick leave (*n* = 252, 8.9%) and annual household income (*n* = 334, 11.7%) had the most missing data. Because of missing data in different cells, the GLMs included 2,800 observations. All analyses were performed in 2021 on Stata version 15.1 (StataCorp, College Station, TX).

## Results

### Descriptive characteristics

Table 1 presents the descriptive characteristics of the sample. A majority of respondents were women (55.7%), between 40 and 59 years of age (56.5%), non-Hispanic whites (93.0%), had a college education (60.4%), were married or partnered (73.8%) and lived in suburban areas (55.7%). There was an even distribution of respondents in each of the four U.S. regions. Compared to non-essential workers, essential workers were significantly more likely to be male, older, white, married, without a college education, and living in rural areas.

**Table 1:** Descriptive characteristics of 2,845 working adults during the COVID-19 pandemic in the US, April 2020

**Essential worker**				
	**Total** **(*n* = 2845)**	**No** **(*n* = 1027)**	**Yes** **(*n* = 1818)**	***p*-value**^**a**^
**SOCIO-DEMOGRAPHIC FACTORS**				
**Female, n (%)**	1585 (55.7)	641 (62.4)	944 (51.9)	<0.001
**Age group, n (%)**				0.014
18-39	566 (19.9)	234 (22.8)	332 (18.3)	
40-59	1607 (56.5)	562 (54.7)	1045 (57.5)	
60+	672 (23.6)	231 (22.5)	441 (24.3)	
**White race, n (%)**	2647 (93.0)	939 (91.4)	1708 (93.9)	0.011
**Some college or less, n (%)**	1127 (39.6)	200 (19.5)	927 (51.0)	<0.001
**Married/partnered, n (%)**	2099 (73.8)	780 (75.9)	1312 (72.3)	0.048
**Residence type, n (%)**				<0.001
Rural	830 (29.2)	241 (23.5)	589 (32.4)	
Suburban	1584 (55.7)	615 (59.9)	969 (53.3)	
Urban	431 (15.1)	171 (16.7)	260 (14.3)	
**U.S. Region, n (%)**				0.262
Northeast	793 (27.9)	299 (29.1)	494 (27.2)	
Midwest	771 (27.1)	257 (25.0)	514 (28.3)	
South	747 (26.3)	270 (26.3)	477 (26.2)	
West	534 (18.8)	201 (19.6)	333 (18.3)	
**ECONOMIC PRECARITY**				
**Annual household income, n (%)**				<0.001
90,000 and over	1215 (42.7)	533 (51.9)	682 (37.5)	
50,000-<90,000	759 (26.7)	253 (24.6)	506 (27.8)	
<50,000	537 (18.9)	124 (12.1)	413 (22.7)	
**Variable income, n (%)**	1563 (54.9)	346 (33.7)	1217 (66.9)	<0.001
**Employer does not offer paid sick leave, n (%)**	701 (24.6)	149 (14.5)	552 (30.4)	<0.001
**No health insurance, n (%)**	139 (4.9)	35 (3.4)	104 (5.7)	0.006
**Lost income due to COVID-19, n (%)**	1116 (39.2)	354 (34.5)	762 (41.9)	<0.001
**Is food insecure, n (%)**	349 (12.3)	77 (7.5)	272 (15.0)	<0.001
**KNOWLEDGE**				
Stopping going to school/work protective, n (%)	2357 (82.8)	935 (91.0)	1422 (78.2)	<0.001
Washing hands frequently with soap and water protective, n (%)	2837 (99.7)	1026 (99.9)	1811 (99.6)	0.153
**COVID-19 THREAT PERCEPTION**				
Perceived risk, M (SD)	5.2 (0.2)	5.1 (0.5)	5.2 (0.3)	0.134
Perceived severity, M (SD)	5.5 (0.2)	5.6 (0.5)	5.4 (0.3)	0.006
**SELF-EFFICACY**				
Can't afford to quarantine, n (%)	821 (28.9)	153 (14.9)	668 (36.7)	<0.001
**ENDORSED PREVENTIVE BEHAVIORS**				
Avoided leaving home, n (%)	2180 (76.6)	904 (88.0)	1276 (70.2)	<0.001
More hand-sanitizer use and/or hand-washing, n (%)	2639 (92.8)	1004 (97.8)	1635 (89.9)	<0.001
Cleaned/disinfected more, n (%)	977 (34.3)	350 (34.1)	627 (34.5)	0.826
Avoided seeking medical care, n (%)	1827 (64.2)	726 (70.7)	1101 (60.6)	<0.001

^*a*^*p*-values calculated based on Pearson’s chi-squared tests for proportions or t-tests for mean differences. Fishers’ exact was used when any cell had *n* < 15. Some columns may not add to 100% due to rounding

**Fig. 2:**
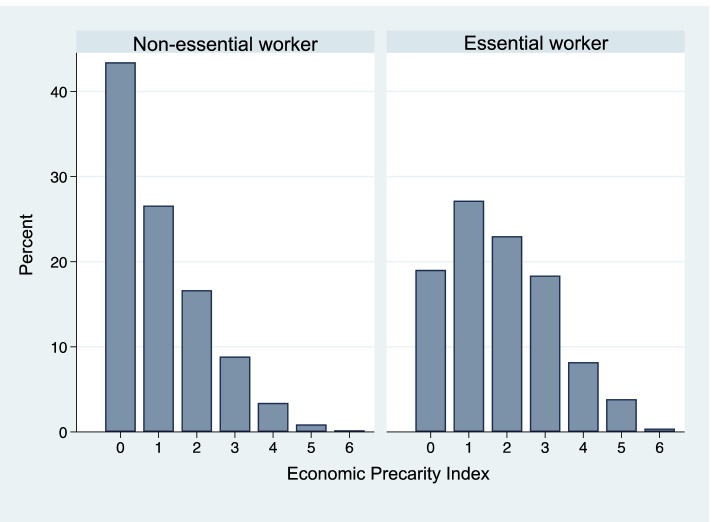
Distribution of economic precarity factors by essential worker status

In terms of income and employment conditions, more than half of respondents earned an annual household income of less than US$90,000 (57.3%) and did not have a fixed income (54.9%), while the majority had paid sick leave (75.4%), had health insurance (95.1%), did not lose income due to COVID-19 (60.8%), and were not food insecure (87.7%). All of these economic precarity indicators were correlated at *p*<0.0001 (Table S1). Notably, among those with no paid sick leave, 84.5% had variable income, and 66.3% lost income due to COVID-19. Overall, 72.0% of respondents had at least one economic precarity factor, while nearly a quarter (24.5%) had three or more. Essential workers had markedly more precarious employment conditions compared to non-essential workers; they were significantly less likely to earn ≥US$90,000 annually, with 22.7% earning less than US$50,000 a year, more likely to have variable income (66.9% *vs.* 33.7%), and more likely to not have health insurance (5.7% *vs.* 3.4%). Close to one-third of essential workers did not have paid sick leave (30.4%), 41.9% had lost income due to COVID-19, and 15% were food insecure. Overall, 81.0% of essential workers had at least one economic precarity factor (Fig. 2).

Knowledge of COVID-19 protective behaviors was high. Overall, 82.8% of respondents identified not going to work or school as a protective behavior, and almost all (99.7%) endorsed that frequent handwashing and handsanitizing were protective against COVID-19 infection. While there were no significant differences concerning these personal hygiene behaviors, a smaller proportion of essential workers (78.2%) endorsed not going to work or school as a protective behavior, compared to those who could work from home (91.0%).

In terms of perceived threat of COVID-19, respondents reported an average risk [mean (M) = 5.2, standard deviation (SD) = 2.3] and severity (M = 5.5, SD = 2.4). While there was no difference in perceived risk between the two groups, essential workers perceived COVID-19 as less severe than non-essential workers.

Engagement in COVID-19 protective behaviors was high in the sample. Increase in hand hygiene was almost universally endorsed (92.8%), and a large proportion of respondents avoided leaving home (76.6%) and seeking medical or dental care for non-COVID-19 health concerns (64.2%). A small proportion of respondents engaged in cleaning and disinfecting more (34.3%). As can be seen in Table 1, except for cleaning and disinfecting more, essential workers were significantly less likely to engage in any protective behavior, more likely to leave home, and less likely to observe personal hygiene-related protective behaviors than non-essential workers.

### COVID-19 protective behaviors, HBM, and economic precarity

**Fig. 3:**
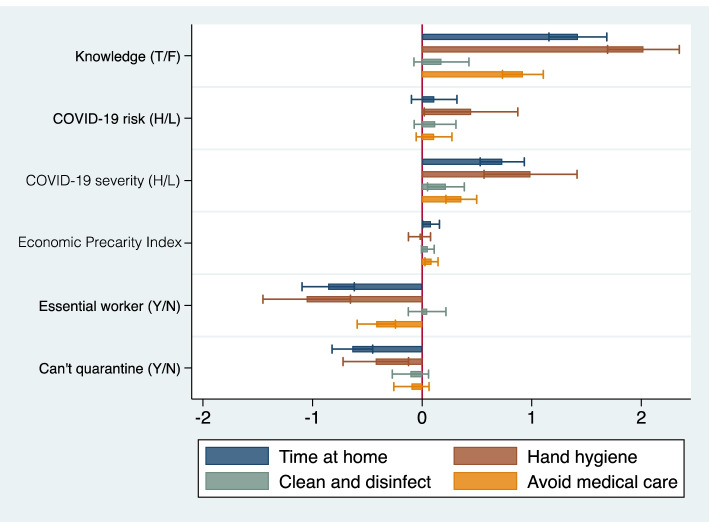
Odds of engaging in a COVID-19 preventive behavior by HBM construct and economic precarity

Tables 2, 3, 4 and 5 present the GLM results, with each table dedicated to a COVID-19 protective behavior. Women were more likely than men to engage in all protective behaviors, except avoiding seeking medical or dental care; adjusted odds ratios (AORs) ranged from 1.9 (95% CI: 1.6 - 2.3) for avoiding leaving home to 1.3 (95 % CI: 1.1 - 1.5) for cleaning and disinfecting more. Respondents with no college education were less likely to avoid leaving home and to engage in handwashing than those with a college degree; however, the association was attenuated when entering the economic vulnerability factors in the model (Tables 2 and 3, change from Model 3 to 4). Middle-aged respondents were more likely to clean and disinfect more (AOR = 1.4, 95% CI: 1.1 - 1.7) than those under 40; but there were no significant differences by age group for the other COVID-19 protective behaviors. Knowing that not going to work or school was protective of COVID-19 infection was, as expected, associated with avoiding leaving home (AOR = 3.6, 95% CI: 2.7 - 4.7), as well as with increased hand hygiene (AOR = 6.0, 95% CI: 4.3 - 8.3) and avoiding seeking medical or dental care for other health concerns, which also necessitates leaving home (AOR = 2.2, 95% CI: 1.9 - 2.7), but not with cleaning and disinfecting more. Greater COVID-19 perceived risk and severity were associated with higher odds of engaging in all protective behaviors, with one exception; the odds of avoiding medical or dental care for other health concerns were positively associated with perceived severity but not with perceived COVID-19 risk. The most robust associations were between perceived severity and avoiding leaving home (AOR = 1.2, 95% CI: 1.1 - 1.3) and increased hand hygiene (AOR = 1.3, 95% CI: 1.2 - 1.4). However, being an essential worker (AOR = 0.4, 95% CI: 0.3 - 0.5) and not being able to afford to quarantine (AOR = 0.5, 95% CI: 0.4 - 0.6), but not economic precarity, were associated with lower odds of staying at home, irrespective of the level of knowledge and perceived threat of COVID-19. Figure 3 presents a graphic illustration of the AORs and 95% CIs.

**Table 2:** Factors associated with avoiding leaving home except to seek food or medicines during the COVID-19 pandemic among 2800 employed U.S. adults, April 2020

	Model 1	Model 2	Model 3	Model 4
	AOR (95% CI)	AOR (95% CI)	AOR (95% CI)	AOR (95% CI)
**SOCIO-DEMOGRAPHIC FACTORS**				
**Female**	**2.5 (2.1, 3.0)**	**2.3 (1.9, 2.7)**	**2.0 (1.7, 2.4)**	**1.9 (1.6, 2.3)**
**Age group**				
18-39	ref	ref	ref	ref
40-59	0.9 (0.7, 1.1)	1.0 (0.7, 1.3)	0.9 (0.7, 1.2)	0.9 (0.7, 1.2)
60+	1.0 (0.8, 1.2)	1.0 (0.8, 1.2)	0.8 (0.6, 1.1)	0.8 (0.6, 1.0)
**White race**	0.9 (0.6, 1.2)	0.8 (0.5, 1.1)	0.7 (0.5, 1.0)	0.8 (0.5, 1.1)
**Some college or less**	**0.7 (0.6, 0.8)**	**0.7 (0.6, 0.9)**	**0.7 (0.6, 0.9)**	0.9 (0.7, 1.2)
**Married**	1.1 (0.9, 1.4)	**1.2 (1.0, 1.5)**	**1.3 (1.1, 1.6)**	1.3 (1.0, 1.6)
**Residence type**				
Rural	ref	ref	ref	ref
Suburban	1.2 (0.9, 1.4)	1.0 (0.8, 1.3)	1.1 (0.8, 1.3)	1.0 (0.8, 1.3)
Urban	**1.5 (1.1, 1.9)**	1.3 (0.98, 1.7)	1.2 (0.9, 1.7)	1.2 (0.9, 1.5)
**KNOWLEDGE**				
Stopping going to school/work protective		**5.8 (4.6, 7.3)**	**4.2 (3.3, 5.4)**	**3.6 (2.7, 4.7)**
**COVID-19 THREAT PERCEPTION**				
Perceived risk			1.0 (0.98, 1.1)	**1.1 (1.0, 1.1)**
Perceived severity			**1.2 (1.1, 1.3)**	**1.2 (1.1, 1.3)**
**ECONOMIC PRECARITY INDEX**				1.1 (1.0, 1.2)
**ESSENTIAL WORKER**				**0.4 (0.3, 0.5)**
**CAN'T AFFORD TO QUARANTINE**				**0.5 (0.4, 0.6)**

*AOR* Adjusted odds ratio, *CI* Confidence interval

**Table 3:** Factors associated with hand-washing and/or sanitizer use during the COVID-19 pandemic among 2800 employed U.S. adults, April 2020

	Model 1	Model 2	Model 3	Model 4
	AOR (95% CI)	AOR (95% CI)	AOR (95% CI)	AOR (95% CI)
**SOCIO-DEMOGRAPHIC FACTORS**				
**Female**	**2.9 (2.0, 4.1)**	**2.3 (1.6, 3.2)**	**1.8 (1.3, 2.5)**	**1.7 (1.2, 2.4)**
**Age group**				
18-39	ref	ref	ref	ref
40-59	0.9 (0.7, 1.4)	1.1 (0.7, 1.6)	1.0 (0.6, 1.5)	1.0 (0.7, 1.6)
60+	1.2 (0.8, 1.9)	1.2 (0.7, 1.9)	1.0 (0.6, 1.6)	1.0 (0.6, 1.6)
**White race**	0.9 (0.5, 1.5)	0.8 (0.4, 1.5)	0.7 (0.4, 1.4)	0.8 (0.4, 1.6)
**Some college or less**	**0.5 (0.3, 0.6)**	**0.6 (0.4, 0.7)**	**0.5 (0.4, 0.7)**	**0.7 (0.5, 0.97)**
**Married**	0.9 (0.7, 1.3)	1.0 (0.7, 1.5)	1.1 (0.8, 1.6)	1.0 (0.7, 1.6)
**Residence type**				
Rural	ref	ref	ref	ref
Suburban	**1.6 (1.2, 2.2)**	**1.5 (1.0, 2.1)**	**1.6 (1.1, 2.2)**	**1.5 (1.0, 2.2)**
Urban	1.4 (0.8, 2.2)	1.1 (0.7, 1.8)	1.0 (0.6, 1.6)	1.0 (0.6, 1.5)
**KNOWLEDGE**				
Stopping going to school/work protective		**11.9 (8.8, 16.0)**	**6.9 (5.0, 9.5)**	**6.0 (4.3, 8.3)**
**COVID-19 THREAT PERCEPTION**				
Perceived risk			**1.1 (1.0, 1.3)**	**1.1 (1.0, 1.3)**
Perceived severity			**1.3 (1.2, 1.4)**	**1.3 (1.2, 1.4)**
**ECONOMIC PRECARITY INDEX**				1.0 (0.9, 1.1)
**ESSENTIAL WORKER**				**0.3 (0.2, 0.5)**
**CAN’T AFFORD TO QUARANTINE**				**0.7 (0.5, 0.9)**

*AOR* Adjusted odds ratio, *CI* Confidence interval deviation

**Table 4:** Factors associated with cleaning and/or disinfecting more during the COVID-19 pandemic among 2800 employed U.S. adults, April 2020

	Model 1	Model 2	Model 3	Model 4
	AOR (95% CI)	AOR (95% CI)	AOR (95% CI)	AOR (95% CI)
**SOCIO-DEMOGRAPHIC FACTORS**				
**Female**	**1.4 (1.2, 1.7)**	**1.4 (1.2, 1.6)**	**1.3 (1.1, 1.5)**	**1.3 (1.1, 1.5)**
**Age group**				
18-39	ref	ref	ref	ref
40-59	**1.3 (1.1, 1.7)**	**1.3 (1.1, 1.7)**	**1.4 (1.1, 1.7)**	**1.4 (1.1, 1.7)**
60+	1.1 (0.9, 1.4)	1.1 (0.9, 1.4)	1.1 (0.9, 1.4)	1.1 (0.9, 1.4)
**White race**	0.9 (0.6, 1.2)	0.9 (0.6, 1.2)	0.8 (0.6, 1.2)	0.8 (0.6, 1.2)
**Some college or less**	1.0 (0.9, 1.3)	1.1 (0.9, 1.3)	1.0 (0.9, 1.3)	1.0 (0.8, 1.2)
**Married**	1.0 (0.8, 1.2)	1.0 (0.8, 1.2)	1.0 (0.8, 1.2)	1.0 (0.8, 1.2)
**Residence type**				
Rural	ref	ref	ref	ref
Suburban	**1.2 (1.0, 1.4)**	1.2 (1.0, 1.4)	1.2 (1.0, 1.4)	1.2 (0.98, 1.4)
Urban	1.2 (0.9, 1.5)	1.1 (0.9, 1.5)	1.1 (0.8, 1.4)	1.1 (0.8, 1.4)
**KNOWLEDGE**				
Stopping going to school/work protective		**1.3 (1.0, 1.6)**	1.1 (0.8, 1.4)	1.1 (0.8, 1.4)
**COVID-19 THREAT PERCEPTION**				
Perceived risk			**1.1 (1.0, 1.1)**	**1.1 (1.0, 1.1)**
Perceived severity			**1.0 (1.0, 1.1)**	**1.0 (1.0, 1.1)**
**ECONOMIC PRECARITY INDEX**				1.0 (0.99, 1.1)
**ESSENTIAL WORKER**				1.0 (0.9, 1.2)
**CAN'T AFFORD TO QUARANTINE**				0.9 (0.8, 1.1)

*AOR* Adjusted odds ratio, *CI* Confidence interval

**Table 5:** Factors associated with avoiding seeking medical care during the COVID-19 pandemic among 2800 employed U.S. adults, April 2020

	Model 1	Model 2	Model 3	Model 4
	AOR (95% CI)	AOR (95% CI)	AOR (95% CI)	AOR (95% CI)
**SOCIO-DEMOGRAPHIC FACTORS**				
**Female**	**1.4 (1.2, 1.6)**	**1.2 (1.0, 1.5)**	1.1 (0.96, 1.3)	1.1 (0.9, 1.3)
**Age group**				
18-39	ref	ref	ref	ref
40-59	1.0 (0.9, 1.2)	1.1 (0.9, 1.2)	1.0 (0.8, 1.2)	1.0 (0.9, 1.2)
60+	1.0 (0.8, 1.3)	1.0 (0.8, 1.3)	0.9 (0.7, 1.2)	0.9 (0.7, 1.2)
**White race**	0.9 (0.7, 1.3)	0.9 (0.6, 1.2)	0.9 (0.6, 1.2)	0.9 (0.6, 1.3)
**Some college or less**	1.1 (0.9, 1.2)	1.2 (1.0, 1.4)	1.1 (0.9, 1.3)	1.2 (0.97, 1.4)
**Married**	1.1 (0.9, 1.2)	1.1 (0.9, 1.3)	1.1 (0.9, 1.3)	1.2 (0.97, 1.4)
**Residence type**				
Rural	ref	ref	ref	ref
Suburban	1.1 (0.9, 1.2)	1.0 (0.9, 1.2)	1.0 (0.9, 1.2)	1.0 (0.9, 1.2)
Urban	1.1 (0.9, 1.4)	1.0 (0.8, 1.2)	1.0 (0.8, 1.2)	0.9 (0.8, 1.2)
**KNOWLEDGE**				
Stopping going to school/work protective		**3.0 (2.5, 3.6)**	**2.4 (2.0, 2.8)**	**2.2 (1.9, 2.7)**
**COVID-19 THREAT PERCEPTION**				
Perceived risk			1.0 (0.98, 1.1)	1.0 (0.99, 1.1)
Perceived severity			**1.1 (1.1, 1.2)**	**1.1 (1.1, 1.2)**
**ECONOMIC PRECARITY INDEX**				**1.1 (1.0, 1.2)**
**ESSENTIAL WORKER**				**0.7 (0.6, 0.8)**
**CAN'T AFFORD TO QUARANTINE**				0.9 (0.8, 1.1)

*AOR* Adjusted odds ratio, *CI* Confidence interval

Each additional point in the economic precarity index was associated with a 10 percent increase in the odds of avoiding seeking medical or dental care for other health concerns (AOR = 1.1, 95% CI: 1.0 - 1.2) but was not associated with the odds of engaging in other protective behaviors. Even after controlling for knowledge, perceived threat, and socio-demographic factors, essential workers were significantly less likely to engage in COVID-19 protective behaviors than non-essential workers, except for cleaning and disinfecting more; AORs ranged from 0.3 (95% CI: 0.2 - 0.5) for increased hand hygiene to 0.7 (95% CI: 0.6 - 0.8) for avoiding seeking medical or dental care for other health concerns. Not being able to afford to quarantine was negatively associated with avoiding leaving home (AOR = 0.5, 95% CI: 0.4 - 0.6) and increased hand hygiene (AOR = 0.7, 95% CI: 0.5 - 0.9), but not with the other two behaviors.

In sensitivity analysis, essential workers were more likely to have higher economic precarity scores and report not being able to afford quarantine. When analyzing each of the economic precarity items’ individuality, essential workers were more likely than non-essential workers to endorse each of the individual economic precarity items, except for not having health insurance (See Table S2).

## Discussion

Consistent with the HBM, findings confirm our first hypothesis that COVID-19 knowledge and threat perception would be positively associated with engagement in protective behaviors among US workers. These findings echo past COVID-19 research showing a positive association between accurate knowledge, perceived threat and likelihood of engaging in protective behaviors, such as handwashing, forgoing of large gatherings, and mask-wearing [31, 32]. However, our findings underscore that structural level factors, specifically employment and economic conditions, are ultimately associated with endorsed behaviors via self-efficacy, as elaborated in our second hypothesis.

We found partial support to our second hypothesis. Whereas we observed that not being able to stay at home negatively impacted respondents’ self-efficacy to follow social distancing directives, we also observed that these factors were negatively associated with hand hygiene. The study design precludes us from determining the causal mechanisms of this association. Whereas we propose that essential workers faced economic barriers to staying at home, we are less clear on the reasons behind lower handwashing despite high knowledge that this behavior was protective. Some have proposed that strong illness-related beliefs may mediate the association of knowledge and adherence to health promoting behaviors. Several studies documented lower adoption of protective behaviors among those with strong beliefs that they would get infected by COVID-19 [31, 33, 34]. Lower hand washing among essential workers who faced daily high-risk of infection may have been a result of perceiving infection as a *fait accompli* and related feelings of futility in engaging in prevention. On the other hand, essential workers may face structural problems to handwashing. Essential work that involves travel to a workplace and regular interaction with coworkers and the public may present barriers to hand hygiene protocols either because of unavailability of running water or soap and/or because jobs are overly demanding, and afford minimal breaks during work hours [35, 36]. Federal and state governments’ response to ensure adequate protection of frontline essential workers has been fragmented and inadequate [37]. While the US Department of Labor provided some guidance on protections such as mandatory personal protective equipment, social distancing requirements, hazard or premium pay, and application of existing or emergency safety standards, it engaged in no meaningful enforcement of these guidelines while weakening the provisions on paid sick leave approved by the US Congress [37]. Furthermore, Congress provisions left millions of frontline essential workers unprotected, as it applied to small employers with less than 500 employees [37]. In addition, workers in the informal sector, which represents about 19% of the US workforce and is made up predominantly of foreign-born and Black and Brown populations, were afforded no or minimal protections to provide an adequate safety net [38]. The fact that other economic precarity indicators, such as no paid sick leave, were not associated with engaging in COVID-19 protective behaviors further supports this hypothesis.

The prevalence of economic precarity indicators was high even in this sample of employed, relatively high-income earners. More than half of respondents in this category did not have fixed income; more than a quarter did not have paid sick leave; and nearly 40% reported having lost income due to COVID-19. Socio-economic vulnerabilities and employment precarities disproportionately burdened essential workers in our sample; for example, 15% of essential workers were classified as food insecure, compared to 10.5% nationwide [39]. Our findings are consistent with national data indicating that essential workers that produce, process, and deliver vital goods and services at their regular workplaces are, overall, more likely to live in low-income households than the US population [37].

Further, not being able to stay at home was negatively associated with most COVID-19 protective behaviors. This points to broader structural barriers for protection against COVID-19 infection and supports that social class is a significant health determinant in the US [40]. It is possible that lower health literacy and trust in official recommendations could explain this phenomenon. Other studies have identified an association between higher health literacy and more positive attitudes towards COVID-19 mitigation measures and the adoption of preventive behaviors [41, 42]. Even before COVID-19, research had established trust as a critical factor in compliance with public health recommendations [43, 44]. However, during COVID-19, trust has been eroded by massive misinformation, which may disproportionately reach those with low SES [45]. Given that knowledge was accounted for in our analysis, literacy is not the entire picture. More research is needed to understand the intertwined drivers and barriers to action, both in terms of employment conditions and trust and understanding of public health messaging.

### Strengths and limitations

Our study is not without limitations. First, the data is cross-sectional; therefore, causality cannot be inferred. Importantly, we cannot assess temporality; therefore, we tacitly posit that COVID-19 threat perception precedes behavior, but the data structure precludes us from confirming this. Further, this manuscript provides a snapshot of the situation at the initial stages of an evolving pandemic with rapidly changing dynamics. Second, as a convenience sample of English-speaking social media users, the sample is not generalizable to the US population in important ways. At the same time, our sample of respondents had representation from every US state and had a balanced distribution by age group and type of residence. The recruitment strategy potentially under-sampled people from lower SES. For example, this survey was not accessible to respondents without access to the Internet. In the US, it is estimated that 70% of people have Facebook accounts, and three-quarters use them daily [46]. It is important to note that 19.6% of US households, predominantly of lower SES, do not have Internet at home [47]. In addition, over 40 million foreign-born adults reside in the US, and 29% of them do not speak English well, which might have precluded them from participating in our study [48]. Our sample of respondents was also overwhelmingly non-Hispanic white; therefore, findings may not be relevant to racial and ethnic minority groups. These factors could have contributed to the under-representation of people from lower SES and/or racial and ethnic minorities, which, in turn, comprise a substantial portion of workers in sectors characterized by precarious employment conditions who have borne the brunt of the COVID-19 pandemic [10, 11, 37]. This sampling biases might have resulted in a bias of estimates towards the null. However, our analysis still showed a significant difference in the economic precarity index and self-efficacy to follow social-distancing behaviors between essential and non-essential workers. Third, our recruitment strategy precluded us from assessing nonresponse bias; response rates may have been affected by factors such as more interest in COVID-19 [49]. Despite these limitations, we chose the current recruitment strategy as it allowed us to rapidly gather data from a large number of participants in the US during the initial phase of the COVID-19 pandemic.

## Conclusions

Our study provides quantitative evidence to expand our understanding of health disparities among the working population with different SES in the US. First, findings from this study reiterate the importance of safety net policies for the working population, such as universal health coverage, paid sick leave, and fixed income, as necessary to engage in health-related protective behaviors. In our study, these emerged as significant factors associated with health disparities. Moreover, they underscore the importance of “equitable distribution” of the impact of health policies, that is the need to consider the underlying difference in disease incidence and/or exposure to risks stemming from SES disparities. US Census data show that the expansion of benefits, such as unemployment insurance benefits and direct cash benefits, may have helped to stave off the worse economic effects of the pandemic on households, despite declines in earnings in 2020 compared to the prior year [50]. Further, the Affordable Care Act has reduced the proportion of uninsured individuals in the US, facilitating access to non-employer-provided and private insurance [51, 52]. This may have mitigated access to care barriers for essential workers and low-income earners with no health insurance benefits [53]. Despite this, our findings underscore that efforts to promote protective behaviors against health threats, such as COVID-19, must have a targeted approach on vulnerable populations, such as essential workers. Otherwise, they will widen SES and health disparities, as people with better access to information and resources will disproportionately benefit from these blanket efforts.

The complex and intertwined relationships between different factors including SES and COVID-19-related protective behaviors, warrant further qualitative analyses to understand the underlying factors and pathways of how various SES components influence the working population’s engagement with protective behaviors and health. Ideally, a combination of multiple policies that address employment conditions, socio-economic vulnerabilities and working conditions should work hand in hand to reduce disparities shown in our study [54].

Findings from this study can contribute to the evidence base supporting national policies that afford a safety net for workers, such as universal health coverage and mandatory paid sick leave, and encourage the private sector to promote secure, quality employment conditions as critical policies to revert the widened socio-economic gap and health disparities in the US since the 1970s, aggravated by the COVID-19 pandemic.

## Supplementary Information

**Additional file 1.**

## Data Availability

The datasets used and/or analyzed during the current study are available from the corresponding author on reasonable request.

## Abbreviations

AOR: Adjusted odds ratio
CDC: US Centers for Disease Control and Prevention
CI: Confidence interval
GLM: Generalized Linear Models
HBM: Health Belief Model
SES: Socio-economic status
US: United States
WHO: World Health Organization

## References

[1] Adler NE, Newman K (2002). Socioeconomic disparities in health: pathways and policies. Health Aff (Millwood)..

[2] Phelan JC, Link BG, Tehranifar P (2010). Social conditions as fundamental causes of health inequalities: theory, evidence, and policy implications. J Health Soc Behav.

[3] Wadhera RK, Wadhera P, Gaba P, Figueroa JF, Joynt Maddox KE, Yeh RW (2020). Variation in COVID-19 hospitalizations and deaths across new york city boroughs. JAMA.

[4] Centers for Disease Control and Prevention. COVID Data Tracker. Atlanta: US Department of Health and Human Services, CDC; 2022. https://covid.cdc.gov/covid-data-tracker.

[5] Koma W, Artiga S, Neuman T, Claxton G, Rae M, Kates K, et al. Low-income and communities of color at higher risk of serious illness if infected with coronavirus. San Francisco: Kaiser Family Foundation; 2020. Available from https://www.kff.org/disparities-policy/issue-brief/low-income-and-communities-of-color-at-higher-risk-of-serious-illness-if-infected-with-coronavirus/.

[6] Montenovo L, Jiang X, Rojas FL, Schmutte IM, Simon KI, Weinberg BA, et al. Determinants of Disparities in Covid-19 Job Losses. Washington, D.C.: National Bureau of Economic Research Working Paper Series, No. 27132; 2020. 10.3386/w2713212.

[7] Clark E, Fredricks K, Woc-Colburn L, Bottazzi ME, Weatherhead J (2020). Disproportionate impact of the COVID-19 pandemic on immigrant communities in the United States. PLoS Negl Trop Dis.

[8] Holman EA, Thompson RR, Garfin DR, Silver RC (2020). The unfolding COVID-19 pandemic: a probability-based, nationally representative study of mental health in the United States. Sci Adv..

[9] Tsai J, Wilson M (2020). COVID-19: a potential public health problem for homeless populations. Lancet Public Health.

[10] Chen YH, Glymour M, Riley A, Balmes J, Duchowny K, Harrison R (2021). Excess mortality associated with the COVID-19 pandemic among Californians 18–65 years of age, by occupational sector and occupation: March through November 2020. PLoS ONE.

[11] Hawkins D, Davis L, Kriebel D (2021). COVID-19 deaths by occupation, Massachusetts, March 1-July 31, 2020. Am J Ind Med.

[12] Mutambudzi M, Niedwiedz C, Macdonald EB, Leyland A, Mair F, Anderson J, et al. Occupation and risk of severe COVID-19: prospective cohort study of 120 075 UK Biobank participants. Occup Environ Med. 2020;78(5):307–14. 10.1136/oemed-2020-106731. Epub ahead of print. Erratum in: Occup Environ Med. 2022 Feb;79(2):e3. PMID: 33298533; PMCID: PMC7611715. 10.1136/oemed-2020-106731 PMC7611715 33298533

[13] Selden TM, Berdahl TA (2020). COVID-19 And racial/ethnic disparities in health risk, employment and household composition. Health Aff (Millwood).

[14] Kalleberg AL (2012). Job quality and precarious work. Work Occup.

[15] Kiersztyn A. Non-standard employment and subjective insecurity: how can we capture job precarity using survey data? In: Kalleberg AL, Vallas SP, editors. Precarious Work. Research in the Sociology of Work. 31: West Yorkshire: Emerald Publishing Limited; 2017. p. 91-122. 10.1108/S0277-283320170000031003.

[16] Jones CL, Jensen JD, Scherr CL, Brown NR, Christy K, Weaver J (2015). The health belief model as an explanatory framework in communication research: exploring parallel, serial, and moderated mediation. Health Commun.

[17] Rosenstock IM, Strecher VJ, Becker MH (1988). Social learning theory and the health belief model. Health Educ Q.

[18] Ali SH, Foreman J, Capasso A, Jones A, Tozan Y, DiClemente RJ (2020). Social media as a recruitment platform for a nationwide online survey of COVID-19 knowledge, beliefs, and practices in the United States: Methodology and feasibility analysis. BMC Med Res Methodol..

[19] Gu LL, Skierkowski D, Florin P, Friend K, Ye Y (2016). Facebook, Twitter, & Qr codes: an exploratory trial examining the feasibility of social media mechanisms for sample recruitment. Comput Hum Behav.

[20] Whitaker C, Stevelink S, Fear N (2017). The use of facebook in recruiting participants for health research purposes: a systematic Review. J Med Internet Res.

[21] Janz NK, Becker MH (1984). The health belief model: a decade later. Health Educ Q.

[22] Painter JE, DiClemente RJ, von Fricken ME (2017). Interest in an Ebola vaccine among a U.S. national sample during the height of the 2014–2016 Ebola outbreak in West Africa. Vaccine..

[23] Painter JE, von Fricken ME, de Viana OMS, DiClemente RJ (2018). Willingness to pay for an Ebola vaccine during the 2014–2016 ebola outbreak in West Africa: Results from a U.S. National sample. Hum Vaccin Immunother..

[24] de Zwart O, Veldhuijzen IK, Elam G, Aro AR, Abraham T, Bishop GD (2009). Perceived threat, risk perception, and efficacy beliefs related to SARS and other (emerging) infectious diseases: results of an international survey. Int J Behav Med.

[25] Najimi A, Golshiri P (2013). Knowledge, beliefs and preventive behaviors regarding Influenza A in students: a test of the health belief model. J Educ Health Promot..

[26] World Health Organization (2020). Survey Tool and Guidance: Rapid, simple, flexible behavioural insights on COVID-19.

[27] U.S. Census Bureau. Census Regions and Divisions of the United States 1984. Silver Hill: U.S. Census Bureau. Available from: https://www2.census.gov/geo/pdfs/maps-data/maps/reference/us_regdiv.pdf.

[28] United States Department of Agriculture. U.S. Household Food Security Survey Module: Six-Item Short Form Economic Research Service. Washington, D.C.: USDA; 2012. Available from: https://www.ers.usda.gov/media/8282/short2012.pdf.

[29] Kaiser Family Foundation. KFF coronavirus poll – March 2020. San Francisco: Kaiser Family Foundation; 2020. Available from: http://files.kff.org/attachment/Topline-KFF-Coronavirus-Poll.pdf.

[30] McCullagh P, Nelder JA (1989). Generalized Linear Models.

[31] Miller LMS, Gee PM, Katz RA (2021). The importance of understanding COVID-19: the role of knowledge in promoting adherence to protective behaviors. Front Public Health..

[32] Clements JM (2020). Knowledge and behaviors toward COVID-19 among US residents during the early days of the pandemic: cross-sectional online questionnaire. JMIR Public Health Surveill.

[33] Jimenez T, Restar A, Helm PJ, Cross RI, Barath D, Arndt J (2020). Fatalism in the context of COVID-19: Perceiving coronavirus as a death sentence predicts reluctance to perform recommended preventive behaviors. SSM Popul Health..

[34] Shahnazi H, Ahmadi-Livani M, Pahlavanzadeh B, Rajabi A, Hamrah MS, Charkazi A (2020). Assessing preventive health behaviors from COVID-19: a cross sectional study with health belief model in Golestan Province, Northern of Iran. Infect Dis Poverty.

[35] Schneider D, Harknett K (2020). Essential and Unprotected: COVID-19-Related Health and Safety Procedures for Service-Sector Workers. Shift Project Research Brief.

[36] Hammonds C, Kerrissey J, Tomaskovic-Devey D (2020). Stressed, Unsafe, and Insecure: Essential Workers Need A New, New Deal. Center for Employment Equity.

[37] Brudney JJ. Forsaken Heroes: COVID-19 and Frontline Essential Workers. Fordham Urb LJ. 2021;48(1). Available from: https://ir.lawnet.fordham.edu/ulj/vol48/iss1/1.

[38] Joassart-Marcelli P (2020). The Pandemic Exposes Dangers of the Informal Economy And It Is Not Just Developing Countries That Are in Trouble. Foreign Affairs.

[39] United States Department of Agriculture. Food Security and Nutrition Assistance 2020. Washington, DC: USDA. updated December 16. Available from: https://www.ers.usda.gov/data-products/ag-and-food-statistics-charting-the-essentials/food-security-and-nutrition-assistance/.

[40] Navarro V (2021). What is Happening in the United States? How Social Classes Influence the Political Life of the Country and its Health and Quality of Life. Int J Health Serv..

[41] Silva MJ, Santos P (2021). The impact of health literacy on knowledge and attitudes towards preventive strategies against COVID-19: a cross-sectional study. Int J Environ Res Public Health..

[42] Patil U, Kostareva U, Hadley M, Manganello JA, Okan O, Dadaczynski K (2021). Health literacy, digital health literacy, and COVID-19 pandemic attitudes and behaviors in U.S. college students: implications for interventions. Int J Environ Res Public Health.

[43] Meredith LS, Eisenman DP, Rhodes H, Ryan G, Long A (2007). Trust influences response to public health messages during a bioterrorist event. J Health Commun.

[44] Fothergill A, Peek LA (2004). Poverty and disasters in the United States: a review of recent sociological findings. Nat Hazards.

[45] Ahmed Siddiqui MY, Mushtaq K, Mohamed MFH, Al Soub H, Hussein Mohamedali MG, Yousaf Z (2020). "Social Media Misinformation"-An Epidemic within the COVID-19 Pandemic. Am J Trop Med Hyg.

[46] Smith A, Anderson M. Social Media Use in 2018. Washington, D.C.: Pew Research Center; 2018. updated March 1, 2018. Available from: https://www.pewresearch.org/internet/2018/03/01/social-media-use-in-2018/.

[47] U.S. Census Bureau. Quick Facts 2019. Silver Hill: U.S. Census Bureau. Available from: https://www.census.gov/quickfacts/fact/table/US/AGE135219#AGE135219.

[48] Gambino CP, Acosta YD, Grieco EM. English-Speaking Ability of the Foreign-Born Population in the United States: 2012. Report Number ACS-26. Washington, D.C.: U.S. Census Bureau; 2014. updated October 8, 2021. Available from: https://www.census.gov/library/publications/2014/acs/acs-26.html.

[49] Fan W, Yan Z (2010). Factors affecting response rates of the web survey: a systematic review. Comput Hum Behav.

[50] US Census Bureau. Income, Poverty and Health Insurance Coverage in the United States: 2020. Washington, D.C.: U.S. Census Bureau; 2021. updated September 21. Available from: https://www.census.gov/newsroom/press-releases/2021/income-poverty-health-insurance-coverage.html.

[51] Alcala HE, Roby DH, Grande DT, McKenna RM, Ortega AN (2018). Insurance Type and Access to Health Care Providers and Appointments Under the Affordable Care Act. Med Care.

[52] Miller S, Wherry LR (2017). Health and Access to Care during the First 2 Years of the ACA Medicaid Expansions. N Engl J Med.

[53] Fronstin P, Woodbury SA. Update: How Many Americans Have Lost Jobs with Employer Health Coverage During the Pandemic? New York: Commonwealth Fund; 2021. Available from: https://www.commonwealthfund.org/blog/2021/update-how-many-americans-have-lost-jobs-employer-health-coverage-during-pandemic.

[54] Templeton A, Guven ST, Hoerst C, Vestergren S, Davidson L, Ballentyne S (2020). Inequalities and identity processes in crises: Recommendations for facilitating safe response to the COVID-19 pandemic. Br J Soc Psychol.

