# Supplementary material for: Employment conditions as barriers to the adoption of COVID-19 mitigation measures: how the COVID-19 pandemic may be deepening health disparities among low-income earners and essential workers in the United States
Source: BMC Public Health. 2022 May 2;22:870. doi: 10.1186/s12889-022-13259-w (PMC9058755; doi:10.1186/s12889-022-13259-w)
Supplement: Supplementary file 1 — Additional file 1. [file 12889_2022_13259_MOESM1_ESM.docx]

**Supplementary material**

| **Table S1. Polychoric correlations among individual measures of the economic precarity index** | | | | | |
| --- | --- | --- | --- | --- | --- |
|  | 1 | 2 | 3 | 4 | 5 |
| Annual household income <$50,000 | 1 |  |  |  |  |
| Variable income | 0.5 | 1 |  |  |  |
| Lost income due to COVID-19 | 0.2 | 0.4 | 1 |  |  |
| Is food insecure | 0.5 | 0.3 | 0.3 | 1 |  |
| No paid sick leave | 0.4 | 0.6 | 0.6 | 0.4 |  |
| No health insurance | 0.3 | 0.4 | 0.3 | 0.3 | 0.5 |
| *Notes:* All at p<0.0001 |  |  |  |  |  |

| **Table S2. Being an essential work and odds of economic precarity** | |
| --- | --- |
|  | AOR (95% CI) |
| **Individual economic precarity variables** |  |
| Annual household income <$50,000 | 1.8 (1.4, 2.3) |
| Variable income | 3.1 (2.7, 3.6) |
| Lost income due to COVID-19 | 1.2 (1.1, 1.4) |
| Is food insecure | 1.9 (1.4, 2.6) |
| No paid sick leave | 2.0 (1.6, 2.5) |
| No health insurance | 1.3 (0.8, 2.1) |
| **Summed job precarity index** | 1.5 (1.4, 1.6) |
| **Can't afford to quarantine** | 2.6 (2.1, 3.1) |
| *Notes:* GLMs control for sex, age, race, marital status, educational attainment and urban residence, and account for clustering by state of residence. AOR = adjusted odds ratio. CI = confidence interval | |
